# Supplementary material for: Content-rich biological network constructed by mining PubMed abstracts
Source: BMC Bioinformatics. 2004 Oct 8;5:147. doi: 10.1186/1471-2105-5-147 (PMC528731; doi:10.1186/1471-2105-5-147)
Supplement: Additional File 2 — The original results of the above study (non-essential files are deleted to keep the file size under the limit set by BMC bioinformatics). [file 1471-2105-5-147-S2.bz2 › chilibotAdditionalFile2/dip40/36ID7539137E131/html/FKBP1A_FRAP1.html]

 


 **FKBP1A** and **FRAP1** 
  
Found 77 abstracts in PubMed, retrieved 40.  
 

 What does Google say? 
 PDF only 
| .edu only 

---

**Interactive relationship** (e.g. stimulation, inhibition, etc)

**Inhibitory relationship**- Rapamycin FKBP12  [ **FKBP1A** ]  inhibited mTOR  [ **FRAP1** ]  activity.  Ref: 12370290 Mol Cell Biol, 2002
- Displacement of rapamycin from FKBP12  [ **FKBP1A** ]  relieves the inhibition of mTOR  [ **FRAP1** ]  and also restores P3k induced transformation.  Ref: 11134523 Proc Natl Acad Sci U S A, 2001
- First, mutations of mTOR  [ **FRAP1** ]  or FKBP12  [ **FKBP1A** ]  prevent rapamycin from binding to mTOR  [ **FRAP1** ] , conferring rapamycin resistance.  Ref: 12030785 Drug Resist Updat, 2001
- In essence, these agents gain function by binding to the immunophilin FK506 binding protein 12  [ **FKBP1A** ]  and the resultant complex inhibits the activity of mTOR  [ **FRAP1** ] .  Ref: 12864941 Clin Breast Cancer, 2003
- Rapamycin inhibits the kinase mTOR  [ **FRAP1** ] , an important regulator of translation, and this inhibition requires binding of the antibiotic to the immunophilin FKBP12  [ **FKBP1A** ] .  Ref: 11134523 Proc Natl Acad Sci U S A, 2001
**Neutral relationship**- RP2 and RP3 have an identical binding affinity linear interaction energy calculation to FKBP12  [ **FKBP1A** ]  as rapamycin but little or no affinity for binding to FRAP  [ **FRAP1** ] .  Ref: 10732978 Bioorg Med Chem, 2000
- Capture of the long lived interaction complex allows the isolation of phage bearing cognate interaction pairs, as we demonstrate for a range of interactions, including Ab antigen pairs and the rapamycin dependent interaction of FKBP 12  [ **FKBP1A** ]  and FRAP  [ **FRAP1** ] .  Ref: 12084913 Proc Natl Acad Sci U S A, 2002
- The FKBP 12  [ **FKBP1A** ]  rapamycin associated protein FRAP  [ **FRAP1** ] , also known as mTOR  [ **FRAP1** ]  and RAFT 1  [ **FRAP1** ]  is a member of the phosphoinositide kinase related kinase family.  Ref: 11707573 Proc Natl Acad Sci U S A, 2001
- FKBP12  [ **FKBP1A** ]  rapamycin associated protein FRAP  [ **FRAP1** ] , also known as mTOR  [ **FRAP1** ]  or RAFT  [ **FRAP1** ]  is the founding member of the phosphatidylinositol kinase related kinase family and functions as a sensor of physiological signals that regulate cell growth.  Ref: 11930000 Proc Natl Acad Sci U S A, 2002
- The rapamycin and FKBP  [ **FKBP1A** ]  target 1 RAFT1  [ **FRAP1** ] , also known as FKBP12  [ **FKBP1A** ]  rapamycin associated protein FRAP  [ **FRAP1** ] , mTOR  [ **FRAP1** ] , regulates the p70S6 kinase p70 S6k and the eukaryotic initiation factor 4E eIF4E binding protein 1 4E BP1.  Ref: 10753870 J Biol Chem, 2000
- The rapamycin and FK506 binding protein FKBP  [ **FKBP1A** ]  target 1 RAFT1  [ **FRAP1** ] , also known as FRAP  [ **FRAP1** ]  is a mammalian homolog of the Saccharomyces cerevisiae target of rapamycin proteins mTOR  [ **FRAP1** ]  that regulates 4E BP1.  Ref: 10698949 EMBO J, 2000
- cDNA sequencing revealed that hSMG 1 encodes a protein of 3031 amino acids containing a conserved kinase domain, a C terminal domain unique to the PIK related kinases and an FKBP12  [ **FKBP1A** ]  rapamycin binding like domain similar to that found in the PIK related kinase mTOR  [ **FRAP1** ] .  Ref: 11331269 J Biol Chem, 2001
- To effect membrane recruitment, a myristoylated rapamycin binding domain from FRAP  [ **FRAP1** ]  mTOR  [ **FRAP1** ] , called M FRB, binds to lipid permeable rapamycin and non bioactive synthetic rapalogs, leading to reversible heterodimerization of M FRB with FKBP  [ **FKBP1A** ]  DeltaPH.Akt.  Ref: 11896462 Gene Ther, 2002

**Non-interactive relationship** (e.g. studied together, co-existance, homology, etc.)

- In complex with FKBP12  [ **FKBP1A** ] , the immunosuppressant rapamycin binds to and inhibits the yeast TOR1 and TOR2 proteins and the mammalian homologue mTOR  [ **FRAP1** ]  FRAP  [ **FRAP1** ]  RAFT1  [ **FRAP1** ] .  Ref: 10436010 Mol Biol Cell, 1999
- Regulation of the rapamycin and FKBP  [ **FKBP1A** ]  target 1 mammalian target of rapamycin  [ **FRAP1** ]  and cap dependent initiation of translation by the c Abl protein tyrosine kinase.  Ref: 10753870 J Biol Chem, 2000
- Syncytium induced p53S15 phosphorylation is mediated by the upregulation activation of mammalian target of rapamycin  [ **FRAP1** ]  mTOR  [ **FRAP1** ] , also called FKBP12  [ **FKBP1A** ]  rapamycin associated protein FRAP  [ **FRAP1** ] , which coimmunoprecipitates with p53.  Ref: 11602639 J Exp Med, 2001
- In complex with its cellular receptor, the FK506 binding protein FKBP12  [ **FKBP1A** ] , rapamycin binds and inhibits the function of the mammalian target of rapamycin  [ **FRAP1** ]  mTOR  [ **FRAP1** ] .  Ref: 12234610 Biochem Pharmacol, 2002
- Rapamycin binds intracellularly to the immunophilin FK506 binding protein 12  [ **FKBP1A** ]  FKBP12  [ **FKBP1A** ] , and the resultant complex inhibits the protein kinase activity of a protein kinase termed mammalian target of rapamycin  [ **FRAP1** ]  mTOR  [ **FRAP1** ] .  Ref: 11426655 Oncogene, 2000
- In this study, we show that FKBP12  [ **FKBP1A** ]  rapamycin associated protein FRAP  [ **FRAP1** ] , also called mTOR  [ **FRAP1** ]  RAFT  [ **FRAP1** ]  interacts with CLIP 170.  Ref: 12231510 EMBO Rep, 2002
- Upon BMP4 treatment, the serine threonine kinase FKBP12  [ **FKBP1A** ]  rapamycin associated protein FRAP  [ **FRAP1** ] , mammalian target of rapamycin  [ **FRAP1** ]  mTOR  [ **FRAP1** ] , associates with Stat3 and facilitates STAT activation.  Ref: 12796477 J Cell Biol, 2003
- We report here that treatment of Jurkat T cells with an inhibitor of protein tyrosine phosphatase, pervanadate, induces the association of 14 3 3tau with a translational control factor, FKBP12  [ **FKBP1A** ]  rapamycin associated protein FRAP  [ **FRAP1** ] , with significant latter s autophosphorylation.  Ref: 10664457 FEBS Lett, 2000
- One such target is p70S6k1 S6K1, a serine threonine kinase which is inactivated by the mTOR  [ **FRAP1** ]  FKBP12  [ **FKBP1A** ]  rapamycin complex, and which has been linked to translational control by virtue of its ability to phosphorylate the ribosomal protein S6.  Ref: 10490847 Oncogene, 1999
- The FK506 FKBP12  [ **FKBP1A** ]  binding domain of the kinase FRAP  [ **FRAP1** ]  FRB forms a classic up down four helical bundle.  Ref: 12175232 J Am Chem Soc, 2002
- A transcription factor cassette responsive to the natural product dimerizer rapamycin was optimized for retroviral delivery by fusing a highly potent chimeric activation domain to the rapamycin binding domain of FKBP  [ **FKBP1A** ]  rapamycin associated protein FRAP  [ **FRAP1** ] .  Ref: 11078518 Proc Natl Acad Sci U S A, 2000
- FKBP12  [ **FKBP1A** ]  rapamycin associated protein FRAP  [ **FRAP1** ]  autophosphorylates at serine 2481 under translationally repressive conditions.  Ref: 10702316 J Biol Chem, 2000
- The FKBP12  [ **FKBP1A** ]  rapamycin associated protein FRAP  [ **FRAP1** ] , also RAFT  [ **FRAP1** ] , mTOR  [ **FRAP1** ]  belongs to a family of phosphatidylinositol kinase related kinases.  Ref: 10702316 J Biol Chem, 2000
- Human immunodeficiency virus 1 envelope glycoprotein complex induced apoptosis involves mammalian target of rapamycin  [ **FRAP1** ]  FKBP12  [ **FKBP1A** ]  rapamycin associated protein mediated p53 phosphorylation.  Ref: 11602639 J Exp Med, 2001
- Such activation of p70 S6K is blocked by pharmacological inhibitors of the PI 3 kinase or the FKBP 12  [ **FKBP1A** ]  rapamycin associated protein mammalian target of rapamycin  [ **FRAP1** ]  FRAP  [ **FRAP1** ]  mTOR  [ **FRAP1** ] .  Ref: 12759354 J Biol Chem< MedlineTA>J Biol Chem, 2001
- FKBP12  [ **FKBP1A** ]  rapamycin associated protein FRAP  [ **FRAP1** ] .  Ref: 11114166 Proc Natl Acad Sci U S A, 2000
- Interestingly, the phosphorylation of Thr 69 and Ser 64 was much more sensitive to inhibition by rapamycin FKBP12  [ **FKBP1A** ]  than the phosphorylation of Thr 36 45, and the phosphorylation of Ser 64 by mTOR  [ **FRAP1** ]  was facilitated by phosphorylation of Thr 36, Thr 45, and Thr 69.  Ref: 10942774 J Biol Chem, 2000
- eIF4G Delta and Delta eIF4G were fused C and N terminally to the FK506 binding protein FKBP  [ **FKBP1A** ]  and the FKBP  [ **FKBP1A** ]  rapamycin binding domain FRB of the human FKBP  [ **FKBP1A** ]  rapamycin associated protein FRAP  [ **FRAP1** ] , respectively.  Ref: 12768627 Biotechnol Bioeng, 2003
- Our objective has been to design biosynthetically available analogues of rapamycin that bind tightly to FKBP12  [ **FKBP1A** ]  but not to FRAP  [ **FRAP1** ] .  Ref: 10732978 Bioorg Med Chem, 2000
- The FKBP12  [ **FKBP1A** ]  rapamycin associated protein FRAP  [ **FRAP1** ]  is a CLIP 170 kinase.  Ref: 12231510 EMBO Rep, 2002
- RAFT1  [ **FRAP1** ]  rapamycin and FKBP12  [ **FKBP1A** ]  target 1.  Ref: 10325225 Science, 1999
- Activation of phosphatidylinositol 3 OH kinase and FKBP12  [ **FKBP1A** ]  rapamycin associated mammalian target of rapamycin  [ **FRAP1** ]  FRAP  [ **FRAP1** ]  mTOR  [ **FRAP1** ]  was observed after the treatment with HGF SF.  Ref: 11352916 J Biol Chem, 2001
- In the full length enzyme these sites are also acutely sensitive to the action of FKBP 12  [ **FKBP1A** ]  rapamycin associated protein FRAP  [ **FRAP1** ] .  Ref: 11684675 J Biol Chem, 2002
- The mTOR  [ **FRAP1** ]  FKBP12  [ **FKBP1A** ]  rapamycin complex blocks G1 S transition by inhibiting downstream targets essential for cell cycle progression.  Ref: 10490847 Oncogene, 1999
- Rapamycin inhibits the FK506 binding protein 12  [ **FKBP1A** ]  mammalian target of rapamycin  [ **FRAP1** ]  complex and causes cell cycle arrest in G1.  Ref: 12599018 Urol Res, 2003
- The immunosuppressive properties of rapamycin are a result of the formation of the complex FKBP12  [ **FKBP1A** ]  rapamycin FRAP  [ **FRAP1** ] .  Ref: 10732978 Bioorg Med Chem, 2000
- The activity of p70 ribosomal S6 kinase, a downstream target of the FK506 binding protein 12  [ **FKBP1A** ]  rapamycin associated protein kinase FRAP  [ **FRAP1** ]  kinase RAFT1  [ **FRAP1** ] , mTOR  [ **FRAP1** ] , was identified to be 4.8 0.8 fold, and MMP 1 and MMP 3 protein levels 2.4 and 11.5 fold increased upon UVB irradiation compared with mock irradiated controls.  Ref: 10660603 J Biol Chem, 2000
- The signal from DNA damage to activation of these response pathways appears to be mediated by FKBP12  [ **FKBP1A** ]  rapamycin associated protein FRAP  [ **FRAP1** ] , since these phosphorylation events are blocked by rapamycin.  Ref: 12231188 Methods, 2002
- Rapamycins first bind a cyclophilin FKBP12  [ **FKBP1A** ] , and this complex binds and inhibits the function of mTOR  [ **FRAP1** ]  mammalian target of rapamycin  [ **FRAP1** ]  a serine threonine Ser Thr kinase with homology to phosphatidylinositol 3 kinase.  Ref: 12878853 Cancer Biol TherCancer Biol Ther, 2002
- Phosphoinositide 3 kinase PI3K inhibitors and rapamycin, an inhibitor of the kinase FRAP  [ **FRAP1** ]  mTOR  [ **FRAP1** ]  FKBP12  [ **FKBP1A** ]  rapamycin associated protein mammalian target of rapamycin  [ **FRAP1** ] , prevent the serum induced phosphorylation of these residues.  Ref: 10654941 EMBO J, 2000
